# Genetic Association Studies of Age-Related Traits: New Perspectives

**DOI:** 10.20900/agmr20210003

**Published:** 2020-12-28

**Authors:** Alexander M. Kulminski

**Affiliations:** Biodemography of Aging Research Unit, Social Science Research Institute, Duke University, Durham, NC 27708-0408, USA

**Keywords:** genetic association studies, age-related traits, aging, pleiotropy

## Abstract

Understanding the role of genetic factors in non-Mendelian traits characteristic for post-reproductive life, herein referred to as age-related traits, is lagged behind the understanding of the genetic architecture of Mendelian traits. This lag calls for new, more comprehensive approaches in the analyses of age-related traits leveraging their characteristic features. This paper discusses the role of the inherent heterogeneity in genetic predisposition to age-related traits and pleiotropy. It shows that the comprehensive analyses leveraging such heterogeneity can substantially increase the efficiency and accelerate the progress in uncovering genetic predisposition to such traits.

## INTRODUCTION

Clustering of diseases and longevity in families and the estimates of heritability suggests that health, aging, and longevity can have a genetic component. The Human Genome Project was created to gain insights into the genetic architecture of human disease and related traits [1]. Currently, there is substantial progress in the understanding of the genetic architecture of Mendelian traits; the role of genetic factors in complex, especially age-related traits, is lagged [2].

The progress in Mendelian genetics has been accelerated by large-scale genetic association studies. These studies follow the framework of medical genetics, assuming that there are genetic factors (mutations) directly affecting protein function and causing Mendelian traits [3]. Implicitly, such traits are often assumed to be homogeneous. The untargeted approach implemented in such large-scale studies seems to be promising as such studies identified a large number of the disease-associated mutations and suggested promising disease-management and therapeutic strategies [2,4–6].

In contrast to the Mendelian traits, the age-related traits are complex phenotypes, which do not follow a clear pattern of Mendelian inheritance [7]. Genetic predisposition to such traits is complicated by two major factors. The first is an inherent complexity and redundancy of metabolic networks in human organisms adapted to maximize fitness in different environments during evolutionary selection [8–10]. The second is the lack of apparent and direct connections between genetic factors evolutionary adapted to maximize fitness at reproductive age and age–related traits characteristic for post reproductive life [11]. This problem is complicated by recent changes in the human life span [12] and the fitness landscape [13–15]. Biologists view age-related traits as the results of deviation from the evolutionary adapted mechanisms [11]. The field of aging research suggests various hypotheses to explain the relationships between genetic factors and age-related traits. For example, one hypothesis is so-called antagonistic pleiotropy when a genetic variant evolutionary adapted to benefit fitness in earlier life can be adverse for another trait(s) in late life [16,17].

Given these complexities, genetic predisposition to age-related traits becomes inherently complex, even in genetically homogeneous populations, with critical roles of the interplay of genetic and non-genetic factors underlining, particularly, heterogeneity and pleiotropy [9,18–22]. Then, the conventional strategy attempting to identify simple correlations between genetic variants and non-Mendelian traits may not work [23,24]. Different concepts to adapt the conventional strategy to non-Mendelian traits are considered including, for example, the roles of common and rare variants with small effects, structural diversity of the human genome, intricate genetic architectures of complex traits, incomplete penetrance and variable expressivity, and gene-environment interaction [25–28]. Implementation of such concepts requires appropriate approaches adapted to deal with the complex roles of genes in age-related traits.

This grant report provides short summaries of recent papers to illustrate substantial advantages of utilizing comprehensive approaches in the analyses of genetic predisposition to age-related traits. These approaches, implemented as a synthesis of different statistical methods, laid ground for the AG047310 grant focused on: (Aim 1) construction of age-related phenotypes; (Aim 2) identification of genetic predisposition to endophenotypes; (Aim 3) identification of genetic associations with risks of morbidity, disability, and mortality; (Aim 4) elucidating systemic role of the identified genetic variants in health, wellbeing, and survival, and (Aim 5) dissecting biological role of genes for the revealed single nucleotide polymorphisms (SNPs). The emphasis of the grant aims is on comprehensive strategies to dissect the role of the inherent heterogeneity in genetic predisposition to age-related traits and pleiotropy.

## BIOLOGICALLY-PLAUSIBLE HETEROGENEITY IN PREDISPOSITION TO AGE-RELATED TRAITS IN THE *ApoB* GENE

The inherent heterogeneity in genetic predisposition to age-related traits suggests that differences in the associations across different population groups, even if they are genetically homogeneous, can be biologically motivated. Accordingly, unusual, even antagonistic, relationships between the same allele and the same trait in different population groups are biologically plausible [29,30]. Identifying biologically-plausible heterogeneity in genetic predisposition to age-related traits requires comprehensive approaches. An illustrative example was in [31]. Here, I briefly outline the essence of the analyses and promising results.

We, particularly, examined the association of rs693 SNP from the Apolipoprotein B (*ApoB*) gene with several age-related traits in four independent studies, which included subjects of European ancestry from the Framingham Heart Study (FHS), the Atherosclerosis Risk in Communities (ARIC) Study, the Multi-Ethnic Study of Atherosclerosis (MESA), and the Cardiovascular Health Study (CHS). We considered total cholesterol (TC) and high-density lipoprotein cholesterol (HDL-C) as endophenotypes (the concept of endophenotypes was introduced in psychiatry [32]) and myocardial infarction (MI) as a downstream phenotype. Our approach represented a synthesis of several methods implemented using conventional models in genetic association studies. Specifically, first, we examined associations of rs693 with TC, HDL-C, and MI, separately. Then, we evaluated additive associations of rs693 and endophenotypes with MI. Finally, we performed comparative analyses of the associations of rs693 with MI without and with adjustment by endophenotypes in each of the four datasets comprised of 20,748 subjects with 2357 MI events. This comparative analysis is one of the most straightforward methods to examine whether lipids can mediate the rs693-MI association.

Consistently with prior genome-wide association study (GWAS) meta-analyses [33–35], our analysis confirms a robust adverse association of the rs693_A allele with TC (i.e., TC-increasing) in each study. In contrast, the association of rs693_A with MI may not be in line with the association with TC, despite the compelling role of lipids in cardiovascular health [36,37]. Specifically, the rs693_A allele was beneficial against MI in two independent studies, the ARIC and FHS. Controlling for lipids substantially and consistently *strengthened* the beneficial association of rs693_A with MI in these studies. Also, we found that consistently with the adverse relationship between the rs693_A allele and TC, this allele was adversely associated with MI in CHS and MESA. However, despite this consistency, this relationship was not mediated by lipids. These antagonistic associations with MI were of about the same effect size but of opposite directions in ARIC/FHS and CHS/MESA. A meta-analysis of the results from all studies following the GWAS strategy showed a non-significant association of rs693_A with MI. In contrast, taking into account antagonistic effects, this association was strong and nearly genome-wide significant.

These results caution against simplistic strategies for gaining profound insights into genetic predisposition to age-related traits.

## ANTAGONISTIC HETEROGENEITY IN PLEIOTROPY

Another challenge is that genetic variants can show pleiotropy, which appears to be wide-spread in human disease and related traits [38]. Pleiotropy is particularly a complex phenomenon in predisposition to non-Mendelian age-related traits [39]. Given an interdependence of human metabolic networks and the evolutionary concerns (see above), the same genetic variant(s) can predispose not only to related but also to seemingly unrelated traits in heterogeneous manner [9,21,40]. To examine the role of the inherent heterogeneity in pleiotropic predisposition to age-related phenotypes, we performed a genome-wide pleiotropic meta-analysis of 20 traits [41]. These traits included 12 quantitative markers (e.g., lipids), seven diseases (e.g., diabetes), and death. We used a sample of 33,431 individuals of European ancestry from five longitudinal studies (ARIC, CHS, FHS, MESA, and the Health and Retirement Study).

The analyses were performed in two stages. The first stage was designed to perform univariate GWAS by associating each SNP with each trait in each study separately. This analysis was enhanced by leveraging longitudinal information on traits. The univariate study-specific analysis provides the estimates of the effects and their significances, so for each SNP, we have a 20 × 5 table, which includes 20 estimates in each of 5 studies.

The second stage was to combine these statistics and to perform comparative analysis. In [41], we selected 1000 promising SNPs; the results of the analyses of the larger number of SNPs from the Candidate gene Association Resource were presented in [42]. To perform a pleiotropic meta-analysis, we need to combine statistics for all estimates for each SNP. It can be done in two ways. One is to meta-analyze the results first across studies and then across traits. The other is to meta-analyze the results first across traits and then across studies. Because we assume that these 100 (=20 × 5) genetic associations can be biologically plausible, we used several tests that take and do not take into account potential correlations between the effect statistics or traits.

A conventional meta-analysis across studies for each trait separately identified only 18 trait-associated SNPs. Of them, 16 SNPs replicated previously reported associations, primarily with lipids. This result is not surprising as the sample of 33,431 subjects is modest compared to the consortia-based GWAS.

In contrast, our pleiotropic meta-analyses identified a large number of 124 SNPs with pleiotropic associations. The vast majority of these SNPs, 93% (115 of 124), were novel. They attained genome-wide significance by combining associations with multiple traits, which did not reach that significance in the univariate meta-analysis. Our analysis showed that the associations for 94% (108 of 115) novel pleiotropic SNPs, were strongly affected by the inherent heterogeneity in predisposition to age-related traits. This strong effect was seen as a phenomenon of antagonistic heterogeneity (also seen in [31]) when the effect directions for SNP-trait associations were not aligned with the directions of correlation between traits [41,42]. For example, despite the direct correlation between TC and low-density lipoprotein cholesterol (LDL-C) (e.g., increased level of TC is correlated with an increased level of LDL-C), the effect directions for the associations of the same allele with TC and LDL-C were of opposite signs. This misalignment between the effect directions and correlation cannot be observed in the case of homogeneous genetic predisposition to such traits because homogeneity implies the same relationship between genetic factors and traits in the entire sample, and it’s any subsample. The association signals become stronger when this misalignment is taken into account.

The results of our analyses strongly support the pivotal role of the inherent heterogeneity in genetic predisposition to age-related traits in pleiotropy. Dissecting such heterogeneity can substantially increase the efficiency of the analyses and provide novel insights into the genetic architecture of such traits.

## CONCLUSIONS

The progress in genetic association studies of various traits is mostly attributed to persistent efforts of big consortia on the national and international scale. Since the inception, however, the strategy of large-scale genetic association studies remains mainly unchanged regardless of the type of the analyzed traits. The age-related traits characteristic of health decline, the aging process, and longevity require more comprehensive methodology to gain insights into an inherent complexity of mechanisms involved in their genetic and non-genetic regulation. The main advantage of the strategy outlined in this grant report is its ability to improve the scientific understanding of genetic predisposition to age-related traits by dissecting substantial fraction of the inherently heterogeneous architecture of such traits. This improvement, however, comes with the price as implementation of such a strategy requires data over the humans’ life course and effort consuming analytical work. Nevertheless, this grant report shows highly promising potential of such comprehensive strategies in studies of genetic architecture of age-related traits and indicates that current discoveries from the large-scale studies are likely just “the tip of the iceberg”.
